# Plasticity in the sensorimotor cortex induced by Music-supported therapy in stroke patients: a TMS study

**DOI:** 10.3389/fnhum.2013.00494

**Published:** 2013-09-03

**Authors:** Jennifer Grau-Sánchez, Julià L. Amengual, Nuria Rojo, Misericordia Veciana de las Heras, Jordi Montero, Francisco Rubio, Eckart Altenmüller, Thomas F. Münte, Antoni Rodríguez-Fornells

**Affiliations:** ^1^Cognition and Brain Plasticity Group, Bellvitge Biomedical Research Institute - IDIBELLBarcelona, Spain; ^2^Neurology Section, Hospital Universitari de Bellvitge, Bellvitge Biomedical Research Institute - IDIBELLBarcelona, Spain; ^3^Institute of Music Physiology and Musicians' Medicine, Hannover University of Music, Drama and MediaHannover, Germany; ^4^Department of Neurology, University of LübeckLübeck, Germany; ^5^Department of Basic Psychology, University of BarcelonaBarcelona, Spain; ^6^Catalan Institution for Research and Advanced Studies, ICREABarcelona, Spain

**Keywords:** stroke, music-supported therapy, music, plasticity, transcranial magnetic stimulation

## Abstract

Playing a musical instrument demands the engagement of different neural systems. Recent studies about the musician's brain and musical training highlight that this activity requires the close interaction between motor and somatosensory systems. Moreover, neuroplastic changes have been reported in motor-related areas after short and long-term musical training. Because of its capacity to promote neuroplastic changes, music has been used in the context of stroke neurorehabilitation. The majority of patients suffering from a stroke have motor impairments, preventing them to live independently. Thus, there is an increasing demand for effective restorative interventions for neurological deficits. Music-supported Therapy (MST) has been recently developed to restore motor deficits. We report data of a selected sample of stroke patients who have been enrolled in a MST program (1 month intense music learning). Prior to and after the therapy, patients were evaluated with different behavioral motor tests. Transcranial Magnetic Stimulation (TMS) was applied to evaluate changes in the sensorimotor representations underlying the motor gains observed. Several parameters of excitability of the motor cortex were assessed as well as the cortical somatotopic representation of a muscle in the affected hand. Our results revealed that participants obtained significant motor improvements in the paretic hand and those changes were accompanied by changes in the excitability of the motor cortex. Thus, MST leads to neuroplastic changes in the motor cortex of stroke patients which may explain its efficacy.

## Introduction

Stroke represents a major cause of death and the most important cause of acquired disability in adults of developed countries (World Health Organization, 2003). In stroke survivors, motor deficits are present in a majority of patients (Rathore et al., 2002), leading to limitations in the participation of activities of daily living and preventing patients to live independently. For this reason, restoration of motor deficits is the target of many different therapies (Langhorne et al., 2011).

Usually, the rehabilitation process of motor impairments comprises different stages. At the beginning, motor function is assessed through domain-specific measures in order to set goals with the patient. Subsequently, therapeutic interventions are provided and, finally, reassessment is performed to ensure that motor improvements have been achieved (Warlow et al., 2008). In practice, this process is not always evidence-based but many times guided by the practitioner's expertise. Thus, there is a necessity to investigate effective motor rehabilitation therapies to provide evidence for clinicians (Taub et al., 2002; Cramer et al., 2011; Langhorne et al., 2011).

Besides their clinical efficacy, rehabilitation techniques may be validated by evidence for neuroplasticity which is defined as the capacity of the central nervous system to reorganize its structure, function and connections in response to internal and external constraints and goals during learning, development or after injury (Kolb and Whishaw, 1998; Cramer et al., 2011). Neuroplasticity may be induced due to therapy as behavior can lead to a reorganization of representational maps (Nudo et al., 1996; Muellbacher et al., 2002) as well as intra- and interhemispheric changes and balance (Chollet et al., 1991; Murase et al., 2004; Grefkes et al., 2008).

One of the most studied rehabilitation techniques is the Constraint-Induced Therapy (CIT) (Taub et al., 1993), which comprises the forced use of the paretic extremity for many hours a day by restricting movement of the healthy extremity in combination with shaping techniques. Studies in subacute and chronic patients have shown improvements in motor function that are accompanied with cortical reorganization of motor regions evidenced by Transcranial Magnetic Stimulation (TMS) (Taub et al., 1993; Liepert et al., 1998). For example, Liepert et al. (2000) reported an expansion of the contralateral cortical area responsible for arm movements after the application of CIT. It has been suggested that the success of this therapy may rely on repetitive massed practice of movements performed with the affected extremity overcoming its learned non-use. Notice that learning processes feature prominently not only in neurorehabilitation (Krakauer, 2006) but also in the development of the motor deficits themselves. For example, patients with a motor deficit of the right hand will learn to perform movements predominantly with the (usually non-dominant) left hand. At the same time, this may lead to additional learned non-use of the right hand. It is important to develop new therapeutic strategies to overcome the learned non-use of the affected side, paying special attention on how to perform specific movements. A way to achieve this goal could be through techniques where there is a specific training for patients in activities that could represent the acquisition of new motor skills that could promote brain plasticity (Dayan and Cohen, 2011). During motor skill learning, massive practice of movements can reduce kinematic and dynamic execution errors (Krakauer et al., 1999; Doyon and Benali, 2005). On the other hand, motor skill training will be more effective if task variability is introduced in the training program. These variations could be related to sensorial cues involved in the training (multimodality) which leads to dynamic sensorimotor readjustments and, consequently, internal motor control models can be created and generalized to other situations (Conditt et al., 1997). In this regard, it has been demonstrated that neuroplasticity could be observed at cortical and subcortical levels due to motor skill learning (Karni et al., 1995; Nudo et al., 1996; Willingham, 1998; Draganski et al., 2004; Dayan and Cohen, 2011; Penhune and Steele, 2012).

One example of a skill involving movements of the hand is musical instrument playing. The presence of music during motor learning posits unique and complex demands for the central nervous system (Zatorre et al., 2007), as playing an instrument requires the integration of multimodal information (auditory, visual, and sensorimotor information) (Pantev and Herholz, 2011). During music performance there are feedback and feedforward interactions between the auditory and premotor areas of the cortex. As in other motor skills, motor, premotor, supplementary motor area (SMA), the cerebellum and the basal ganglia are involved in musical motor performance (Lotze et al., 2003; Meister et al., 2004). In addition, the sound of the instrument processed by the auditory cortex can be used to readjust movements leading to interactions between the auditory and motor systems (Zatorre et al., 2007). Compared to other sensorimotor activities, music learning involves the integrated activity of motor and auditory systems. Furthermore, because of the consequent and consistent auditory feedback (Zatorre, 2003), correction of errors in timing, strength and position of the movement is possible. Studies with functional Magnetic Resonance Imaging (fMRI) exploring professional musicians and non-musicians have demonstrated that musical training leads to structural and functional changes in motor regions of the brain, especially those involving auditory and sensorimotor cerebral networks (Gaser and Schlaug, 2003; Bengtsson et al., 2005; Bangert et al., 2006; Baumann et al., 2007; Hyde et al., 2009; Herholz and Zatorre, 2012; Steele et al., 2013). For instance, in healthy subjects, motor cortex was explored with TMS when participants were trained to play the piano showing an enlargement of the cortical representation of the hand after the training (Pascual-Leone et al., 1995). Therefore, learning to play the piano is an example of a music making activity that requires repetitive massed practice and entails variations in the training task (i.e., movement sequences), involving complex coordination. Moreover, playing the piano engages different regions of the brain and could be associated with structural and functional brain changes. Beyond the plasticity in motor regions associated to music making, studies investigating the effects of music listening as a rehabilitative intervention have revealed improvements in cognition and emotional factors (Särkämö et al., 2008; Särkämö and Soto, 2012). These findings add value to the interventions based on motor learning using music making because their possible impact in other cognitive and emotional domains aside from the expected motor improvements.

Recently, Schneider et al.(2007, 2010) have developed Music-supported therapy (MST) to restore motor function after stroke. In this therapy, patients are trained to play a MIDI piano and/or an electronic drum set that produces piano tones, involving fine and gross movements, respectively. MST has been tested in stroke patients showing improvements in the execution of movements revealed by an increase in the scores of behavioral motor tests after the application of MST (Schneider et al., 2007, 2010; Altenmüller et al., 2009). A recent study about a single chronic stroke patient showed that MST can lead to improvements in motor function after 2 years since stroke. Gains in motor function were accompanied by changes in motor cortex excitability (evaluated using motor mapping TMS) with an expansion of the cortical representation of the hand and by activation changes in fMRI (Rojo et al., 2011; Rodríguez-Fornells et al., 2012). In addition, Amengual et al. (2013) have reported evidence from a group of chronic patients that have been treated with MST. Patients improved their motor function as well as an increase of the excitability of the motor system was encountered. Moreover, gains in motor performance were correlated with changes in the cortical representation of a muscle of the paretic hand.

In the present study, MST was administered to stroke patients with hemiparesis of the upperlimb to restore their motor function. Thus, the aim of the present study is to investigate improvements in motor function in subacute stroke patients and whether this restoration is accompanied by neuroplastic changes in the sensorimotor cortex.

## Methods

### Participants

**Figure 1:**
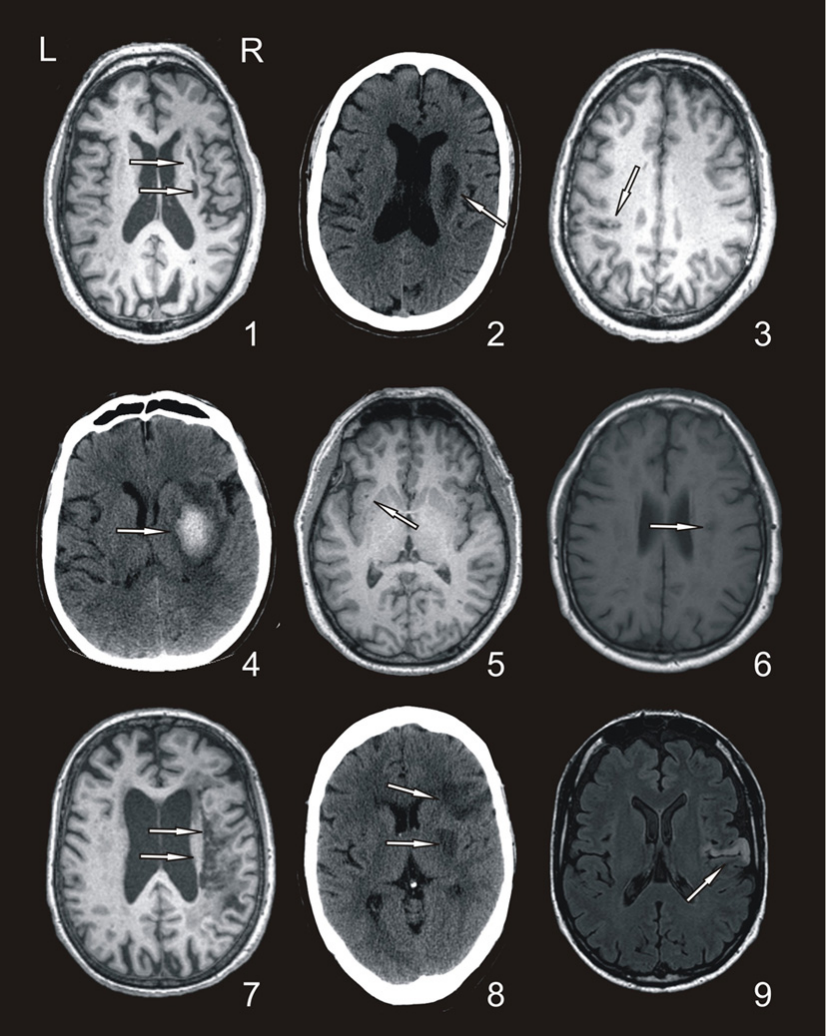
**Representative axial Magnetic Resonance and Computed Tomography images of the patients.** Arrows show the approximate location of each lesion (patient codes are according to the order in Table 1).

Nine right-handed stroke patients with an impairment of motor function of one arm following a stroke participated (3 women, mean age 61.8 ± 9.8 years, years of education 10.8 ± 8). Inclusion criteria were: (1) less than 6 months after stroke, (2) mild-to-moderate paresis of upper extremity, (3) ability to move the affected arm and the index finger without help of the healthy side, (4) Barthel Index score over 50, (5) no major cognitive deficit, (6) no neurological or psychiatric co-morbidity. Table 1 presents individual demographic data and Figure 1 illustrates the lesion for each patient.

**Table 1:** **Demographic data of patients**.

**Subject**	**Age**	Gender^*^	Affected hemisphere^*^	Etiology^*^	**Localization of stroke**	**Time since stroke (months)**	MRCS^*^ PRE	**Barthel index**
1	72	M	R	I	Putamen, globus pallidus and partial damage to the head and body of the caudate nucleus. Anterior and superior portion of the anterior limb of the internal capsule and corona radiata.	3	5−	80
2	63	F	R	I	Lenticular nucleus, body of the caudate nucleus and corona radiata.	2	4−	70
3	59	M	L	I	Cortical and subcortical parietal regions (extreme portion of the superior postcentral gyrus) slightly extended to the surrounding white matter.	1	5−	100
4	63	M	R	H	Lenticular nucleus, internal and external capsule and deep temporal regions.	2	4	100
5	52	M	L	I	External capsule.	2.5	4+	100
6	68	M	R	I	Subcortical damage located in paraventricular regions and semioval center.	4.5	3	100
7	76	F	R	I	Extensive cortical damage to frontotemporoinsular regions extended to subcortical areas including the semioval center and corona radiata.	4	4	60
8	60	F	R	I	Anterior frontal cortex, basal ganglia including the body of the caudate nucleus and the capsular-lenticular region.	5	4	100
9	44	M	R	I	Frontal regions comprising the inferior extreme of the lateral fissure and the superior precentral gyrus.	2.5	4+	100
Mean/SD	61.8/9.8	6M/3F	7R/2L	8I/1H		2.9/1.3	4.1/0.6	88.8/16.7

* M, male; F, female; R, right; L, left; I, ischemic; H, hemorrhagic; MRCS, Medical Research Council Scale for Muscle Strength.

Besides, a matched sample of 9 healthy participants (2 women, mean age 59.3 ± 9.5 years, education 12.2 ± 7 years) composed the control group. Participants in this group were right-handed and without any history of stroke or other neurological or psychiatric disease. They were evaluated in two different time points in order to control repeated imaging testing effects (Johansen-Berg, 2012) and did not receive any training between evaluations.

The study was approved by the Committee of Ethics for Clinical Research of the Hospital Universitari de Bellvitge and fulfilled the standards set by the Declaration of Helsinki. All participants signed an informed consent form explaining the purpose of the study and all procedures.

### Music-supported therapy

During 4 weeks, patients received 20 individual MST sessions of 30 min each. A MIDI-piano and an electronic drum set were used to train fine and gross movements, respectively. For the MIDI-piano, only 8 white keys (G, A, B, C, D, E, F, G′) were used, whereas the electronic drum set comprised 8 pads of 20 cm diameter designated by the numbers 1–8 and programmed to emit piano sounds. During the different sessions, patients had to produce tones, scales and play some simple melodies according to a modular training regime with stepwise increase of complexity (Schneider et al., 2007, 2010). Exercises were adapted to the needs of the individual patient in difficulty and were first shown by the therapist.

### Evaluation

Before and after the MST program, patients were evaluated with regard to their motor function and quality of life. In addition, TMS was performed. Participants in the control group underwent the same evaluation as patients but were not evaluated for quality of life. The second evaluation of the control participants was done between 30 and 40 days after the first assessment.

#### Evaluation of motor function

Motor function was assessed using the Action Research Arm Test (ARAT), Arm Paresis Score, Box and Block Test (BBT) and the Nine Hole pegboard Test (9HTP):

1. ARAT: Patients are assessed for different movements of both upper extremities within four subtests: grasp, grip, pinch, and gross movement which are composed by different items. Scores describe the quality of movement execution with the maximum score being 57 (Carroll, 1965; Lyle, 1981).
2. Arm Paresis Score: Patients are asked to perform 7 movements with either the affected hand or both hands. The maximum score is 7 (Wade et al., 1983).
3. BBT: In this test, patients have to move as many small cubes placed in one compartment of a box to a second compartment within 1 min. The number of moved cubes is scored (Mathiowetz et al., 1985).
4. 9HPT: Patients are asked to place 9 rods (32 mm long, 9 mm diameter) into holes of 10 mm dimeter. Scores are given depending on the time needed to accomplish the task (Parker et al., 1986).

#### Evaluation of quality of life

The Stroke-specific Quality of Life scale (Williams et al., 1999), which is a disease-specific measure of health-related quality of life, was administered to test if MST improved the quality of life of patients. It comprises 12 domains evaluating energy, family roles, language, mobility, mood, personality, self-care, social roles, thinking, upper extremity function, vision, and productivity. Each domain contains different items asking the patient the amount of help required, trouble experienced doing tasks, and the degree of agreement with statements about functioning. The minimum/maximum possible scores are 49/245.

#### Evaluation of cortical excitability

TMS was applied using a 70 mm figure-of-8 coil attached to a Magstim Rapid 2 Stimulator (Magstim Company, Carmathenshire, Wales). The primary motor cortex (M1) was stimulated in a single pulse protocol to elicit motor-evoked potentials (MEPs) from the first dorsal interosseous (FDI). Using surface Ag/AgCl disk electrodes in a belly-tendon montage, electromyographic (EMG) activity from the contralateral FDI was recorded for a total of 700 ms including a 100 ms pre-stimulus window (Medelec Synergy, Oxford Instruments, Pleasantville, NY, USA). The EMG signal was sampled at 5 KHz and band-pass filtered at 1–1000 Hz. Data was exported for off-line analysis using specialized software (Matlab, Mathworks, Natick, MA, USA).

To allow simple identification of stimulation sites, participants wore an elastic cap in which Cz location was marked (international 10/20 EEG positioning system). A grid of 10 × 10 spots was drawn (Cz was centered in the vertex) and the difference from one spot to the other was 1 cm. In this grid there were two perpendicular axes where the *x*-axis is horizontal and the *y*-axis is vertical (two-dimensional, Cartesian coordinate system). Stimulation sites were defined following *x* and *y* axes and becoming coordinates (*x, y*) in the grid (Amengual et al., 2013).

To increase the between-session reliability, a single cap was used per participant in both evaluations. The TMS coil was placed tangentially to the corresponding grid location, with the handle pointing backwards (in a lateral to medial and caudal to rostral position) ~45° lateral from the midline.

Both hemispheres were tested to assess the excitability of the corticospinal pathway using the following parameters: coordinates of Hot Spot, resting motor threshold (RMT), active motor threshold (AMT) (Rossini et al., 1994), cortical silent period (CSP) (Liepert et al., 2005), peak-to-peak amplitude, motor map area and volume, and the coordinates of the center of gravity of the map (CoG_*x*_ and CoG_*y*_) (Wassermann et al., 1992; Byrnes et al., 1999; Amengual et al., 2012, 2013). Below, a further description of each parameter is provided for better understanding.

The Hot Spot is defined by the coordinates yielding the highest MEP in the target muscle. RMT is defined as the lowest stimulus intensity needed to evoke a visible MEP (>50 μV) in 50% of 10 trials from the relaxed target muscle. On the other hand, AMT is defined as the minimum stimulus intensity that produces a visible MEP (>200 μV) in 50% of 10 trials during isometric contraction of the target muscle (Rossini et al., 1994). RMT and AMT are expressed in percentage of maximum stimulation intensity.

The CSP is defined as an interruption of voluntary muscle contraction in response to a TMS pulse (Hallett, 2007). CSP is obtained by applying a suprathreshold TMS pulse (150% of RMT) when the target muscle is preactivated at 10% of its maximum voluntary strength. The EMG typically shows a suppression of the muscle activity which lasts between 100 and 300 ms in healthy subjects. Cortical inhibition is thought to be responsible for the generation of the CSP but spinal inhibitory mechanisms may contribute to the first part of the CSP (Wassermann et al., 2008). The end of the CSP, defined as a return of EMG activity to baseline, is measured in ms.

MEPs peak-to-peak amplitudes, expressed in μV, were obtained at the Hot Spot as the mean of 5 consecutive stimulations at 125% of RMT (Rossini et al., 1994).

To obtain a motor map of the FDI, we recorded 5 MEPs from each different position in the grid at 125% of RMT. The map was generated by plotting peak-to-peak MEP amplitudes at each grid location (Wilson et al., 1993; Byrnes et al., 1999). The area of the map was considered as the number of excitable scalp points (Liepert et al., 1998). As the difference between each spot in the grid was 1 cm, each active spot (MEPs >50 μV) was accounted as 1 cm^2^ of the area of the motor map. The volume of the map was calculated dividing the sum of the amplitudes of the motor map by the area of the map (μV/cm^2^). The center of gravity (CoG) was considered as the amplitude-weighted coordinates of the map (Wassermann et al., 1992). The coordinates of the CoG, named CoG_*x*_ and CoG_*y*_, indicate the position of the spot in the horizontal *x*-axis and the vertical *y*-axis of the grid.

Unfortunately, 3 patients were not eligible for TMS because of severe heart disease (Rossi et al., 2009).

### Analysis

Each patient was paired with a control participant. The control participant's hemisphere corresponding to the affected hemisphere of the patient was considered for comparison. Thus, the hemisphere that is considered as affected in controls is the right with the exception of controls for Patients 1 and 5 who have their lesion in the left hemisphere. Then, for these two controls the hemisphere that is considered as the affected is the left. We performed an exploratory analysis across all parameters in order to identify which TMS variables showed an interactive effect between Group (patients and controls). We used non-parametric test due to the reduced sample size. To this aim, we computed the difference between both evaluations (Post-MST evaluation minus Pre-MST evaluation) for each parameter and applied the Mann–Whitney *U*-test between controls and patients to this difference. The rationale behind this analysis is that only those measures that are affected or sensitive to the treatment will show differences between groups. Notice that this type of analysis was not carried out for the motor performance measures due to the ceiling effects of the control group in these measures. For the motor assessment and when we found differences between groups in TMS parameters, we measured the significance of change between pre-MST and post-MST evaluation using the Wilcoxon signed-rank test. The statistical significance was set to 0.05. Finally, we computed the magnitude of the effect size (*r*) using the criteria stated by Cohen (1992), where a value of 0.10 is a small effect, a value of 0.30 is a medium effect and 0.50 is a large effect.

## Results

### Evaluation of motor function

Results for the ARAT, Arm Paresis Score, BBT and Nine Hole Pegboard Test are summarized in Table 2. As participants in the control group showed maximal scores at the first evaluation, no improvement can be found in this group. In patients, significant improvements were found for the ARAT overall score (*T* = 0, *p* = 0.008, *r* = −0.62), the Arm Paresis Score (*T* = 0, *p* = 0.038, *r* = −0.48) and the BBT score (*T* = 1.5, *p* = 0.012, *r* = −0.58). No differences were observed between pre- and post-MST in the 9HPT score.

**Table 2:** **Results of the motor tests (Mean, SD)**.

**Motor test**	**Patients**		**Controls**	
	**Pre-MST**	**Post-MST**	**Pre**	**Post**
ARAT^*^^b^	37.7 (21.8)	45.5 (5.35)	57 (0)	57 (0)
Arm paresis score^*^^a^	5 (2.5)	5.7 (1.8)	7 (0)	7 (0)
BBT^*^^b^	28.4 (19.5)	33.7 (23.09)	62.2 (10)	68 (8.6)
9HPT	4.7 (4.1)	4.7 (3.8)	9 (0)	9 (0)

a Medium to large effect size;

b Large effect size;

* p < 0.05.

### Evaluation of quality of life

In patients the score of the Stroke-specific Quality of Life scale improved from 160 (±32.5) to 194 (±38.2) (*T* = 5, *p* = 0.038, *r* = −0.48).

### Evaluation of cortical excitability

As stated above, only six patients were assessed in the TMS evaluation. In addition, during the pre-therapy evaluation, one subject did not show MEP responses for the FDI of the affected hand. However, MEP responses for this muscle were observed after the therapy. Consequently, only motor thresholds were considered for this subject as a dependent variable of the affected hemisphere for the statistical analysis. It was not possible to perform the rest of the measurements in this subject. Table 3 shows values of the parameters assessed in each group and hemisphere across time.

**Table 3:** **TMS measurements (Mean/SD)**.

	**Affected hemisphere**				**Unaffected hemisphere**			
	**Patients**		**Controls**		**Patients**		**Controls**	
	**Pre-MST**	**Post-MST**	**Pre**	**Post**	**Pre-MST**	**Post-MST**	**Pre**	**Post**
RMT	69.5	66.8	70.7	71.6	54.3	56.1	66	68
	(17.6)	(15.7)	(12.6)	(10.9)	(13)	(15.1)	(12.1)	(10.6)
AMT	62.1	56.1	53.4	55.4	46.3	44	48.6	50
	(20.3)	(15.1)	(11.7)	(11.5)	(9.7)	(10.6)	(11.7)	(9.4)
CSP	306	377	212.3	219.8	217.3	215.5	232.8	241.6
	(168.3)	(185.6)	(59.6)	(51.9)	(62.1)	(59.2)	(55)	(46.3)
MEP amplitude	389.4	361.8	1161.2	996.6	870.8	1114.8	1102.5	1173
	(238.4)	(296.7)	(919.6)	(876)	(786.8)	(1321.2)	(876)	(1293.9)
Mapping area	18.4	20	18.8	15	15.3	16.3	15.7	16.1
	(6.1)	(7.5)	(5.4)	(5.2)	(5.3)	(6.5)	(5.3)	(5.7)
Mapping volume	9.6	10.9	7.6	6.6	7	7	6.8	6.8
	(4.8)	(5.9)	(1.7)	(1.9)	(2.4)	(3.7)	(1.8)	(2.3)
CoG_*x*_	6.2	6.1	5.5	5.2	5.8	5.8	6.4	6.4
	(1.4)	(1.3)	(0.6)	(0.4)	(0.7)	(0.6)	(0.8)	(0.9)
CoG_*y*_	−1.6	−0.7	−1.6	−1.6	−1.2	−0.4	−1.6	−1.6
	(1)	(1.1)	(0.6)	(0.4)	(1.1)	(1.1)	(0.6)	(0.4)

For the RMT and AMT values represent the intensity of stimulation (from 0 to 100%). The CSP is considered in ms and the MEP amplitude in μ V. The area of the map is considered in cm^2^ and volume in μ V/cm^2^. The CoG is expressed in coordinates (CoG_*x*_ for the x-axis and CoG_*y*_ for the y-axis).

A summary of the Mann–Whitney *U*-test comparing differences between groups in each parameter (measurements obtained subtracting post- minus pre-evaluation) is shown in Table 4.

**Table 4:** **Significance of Mann–Whitney *U*-test comparisons between groups for the TMS measurements obtained subtracting post- vs. pre-treatment measurements**.

	**Affected hemisphere**			**Unaffected hemisphere**		
	***U***	***z***	***p***	***U***	***z***	***p***
RMT	17.5	−1.12	0.260	27	0.00	1
AMT	7	−2.36	0.018^*^	14	−1.54	0.122
MEP amplitude	17	−0.73	0.463	21	−0.70	0.480
CSP	16.5	−0.80	0.423	20.5	−0.26	0.789
Mapping area	3	−2.61	0.009^*^	10	−1.68	0.092
Mapping volume	14	−1.13	0.257	14	−1.13	0.257
CoG_*x*_	18	−0.60	0.544	18	−0.60	0.548
CoG_*y*_	6	−2.20	0.028^*^	7	−2.06	0.039^*^

* p < 0.05.

**Figure 2:**
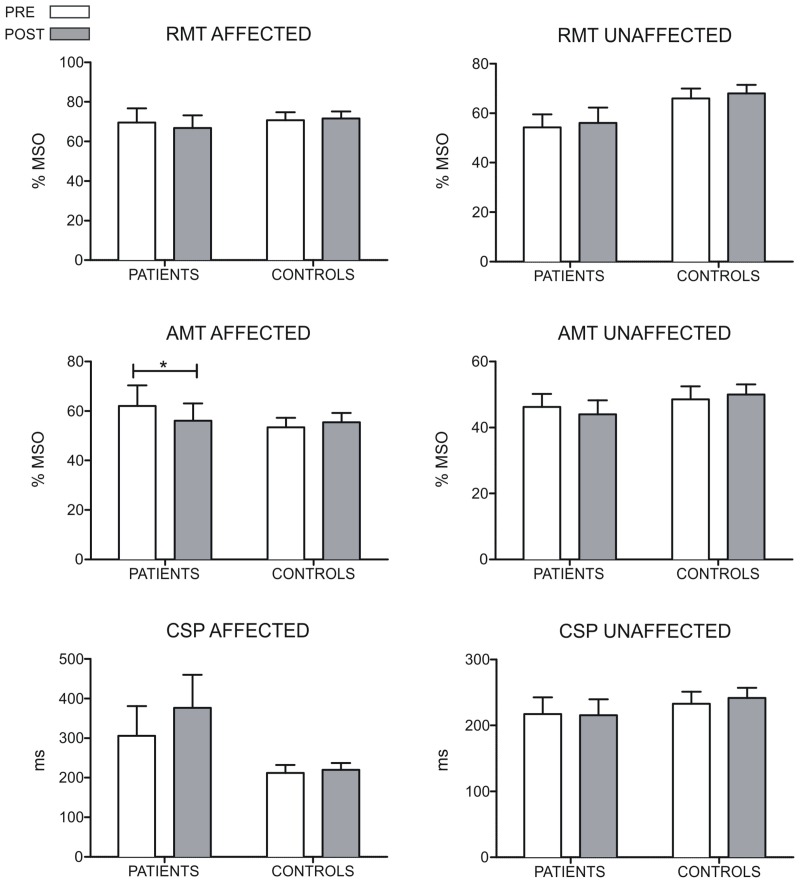
**Results of the RMT, AMT, and CSP measures for both hemispheres of patients and controls.** For the control group, the hemisphere that is compared to the affected in patients is the one corresponding to their matched participant in the patient's group. The RMT and AMT are expressed in percentages of stimulation intensity. The CSP is accounted in *ms*. The AMT of the affected hemisphere decreases in patients after the MST-program (*p* = 0.042). For the RMT and CSP no differences were found in any group (^*^p < 0.05).

We did not find any difference in RMT between groups and across time. However, we found a significant between-group difference in the change of the AMT of the affected hemisphere between both evaluations (*U* = 7, *z* = −2.36, *p* = 0.018, *r* = −0.60). Conversely, we did not find differences between both groups in the change of the AMT in the unaffected hemisphere (*U* = 14, *z* = −1.54, *p* = 1.12, *r* = −0.39). Comparisons in patients between pre-MST and post-MST evaluation revealed a significant reduction of the AMT in the affected hemisphere (*T* = 0, *p* = 0.042, *r* = −0.58), but no changes were observed in the unaffected hemisphere (*T* = 2.25, *p* = 0.207, *r* = −0.36) in patients. No changes in AMT were observed in controls in both hemispheres (*p* > 0.15 for both comparisons). Any difference was found between groups, time and hemispheres for the CSP. Figure 2 shows the results of RMT, AMT, and CSP.

**Figure 3:**
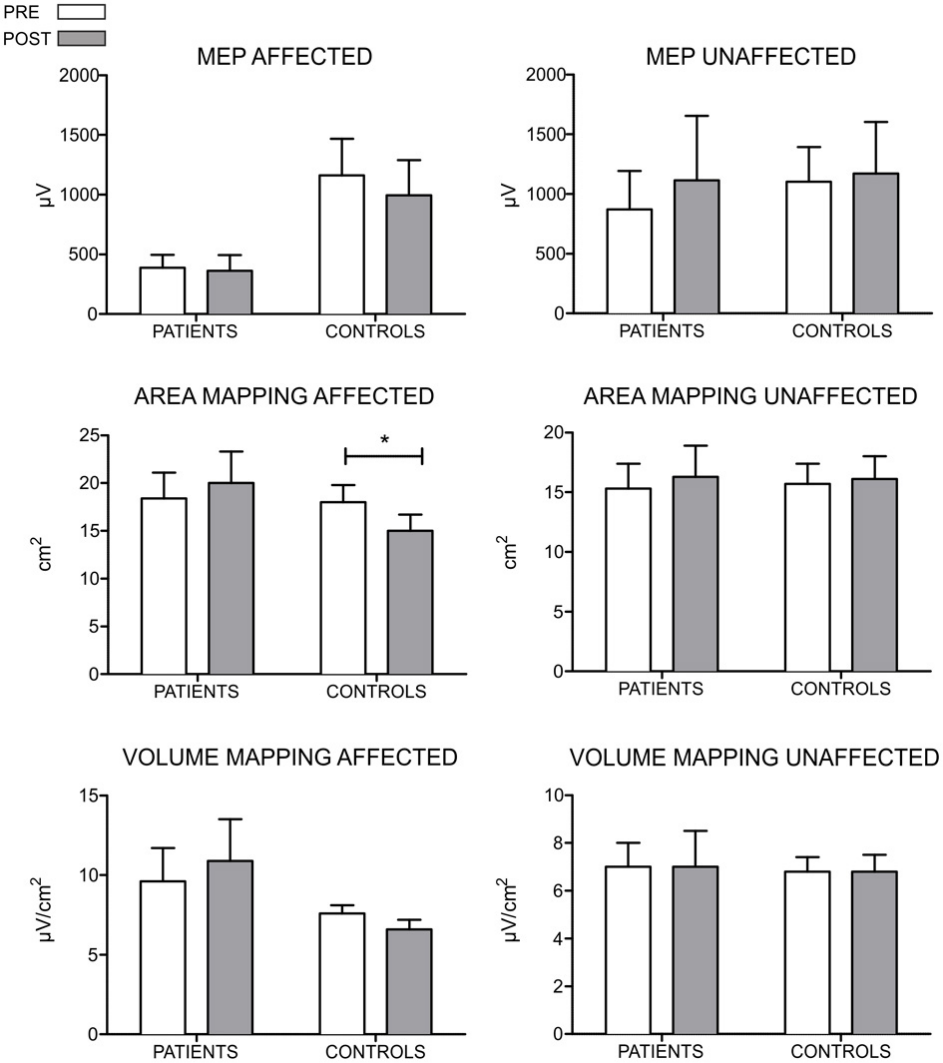
**Results of the MEP amplitude, area, and volume of the TMS motor mapping for both hemispheres of patients and control participants.** For the control group, the hemisphere that is compared to the affected in patients is the one corresponding to their matched participant in the patient's group. The MEP amplitude is expressed in μ*V*, the area in cm^2^ and the volume in μ*V*/ cm^2^. For the MEP amplitude, no differences between groups and across time were encountered. However, control participants seem to show larger amplitude on the elicited MEPs. Regarding to measurements of mapping, no differences were found in the area and volume when comparing groups and across time (^*^p < 0.05).

**Figure 4:**
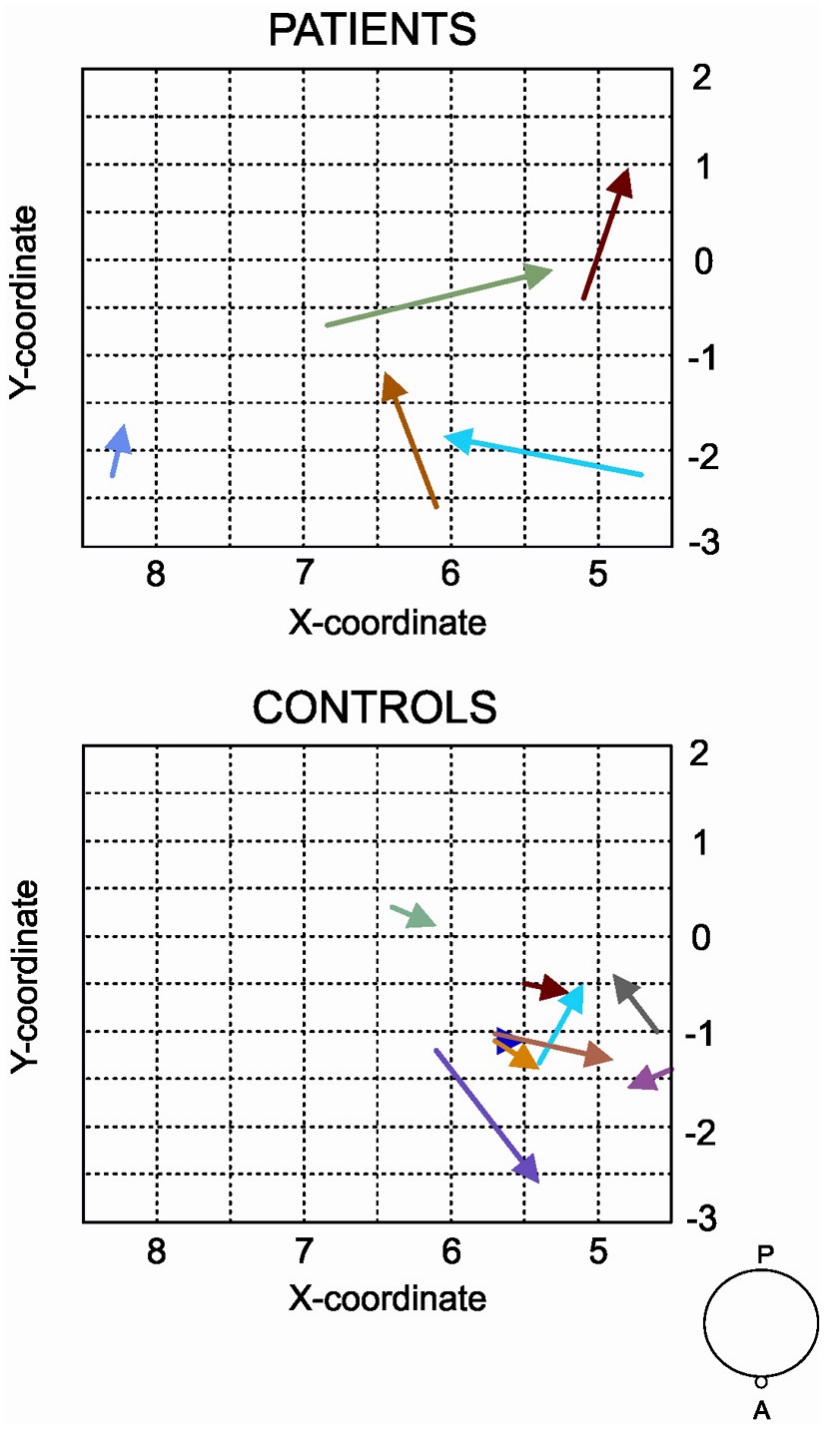
**Displacement of the CoG for the contralateral maps of the FDI after the MST program for the affected hemisphere of patients and controls.** For the control group, the hemisphere that is compared to the affected in patients is the one corresponding to their matched participant in the patient's group. This figure symbolizes the grid where motor maps were drawn and each arrow represents one participant. The beginning of the arrow shows where the CoG (expressed in coordinates *x, y*) was in the pre-MST evaluation and the tip of the arrow indicates the position of the CoG in the post-MST evaluation. We found a significant shift toward more posterior areas in patients after the MST program.

**Figure 5:**
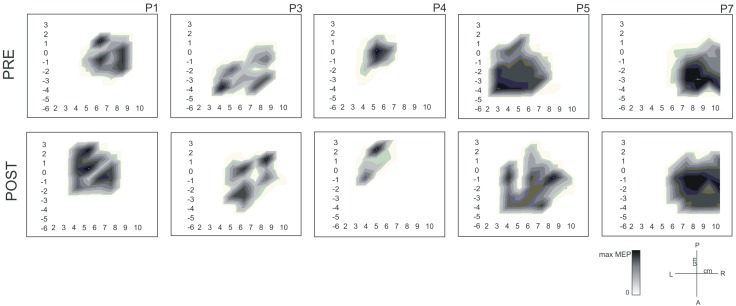
**Cortical motor maps for the first dorsal interosseous (FDI) muscle of the affected hand before and after the MST for 5 patients (P1, P3, P4, P5, and P7).** Values are normalized for each patient according to their maximum MEP amplitude (μ*V*). Moreover, we added units in the part of the manuscript describing the TMS protocol and we improved the definition of each parameter of TMS.

Regarding to the measurements of the motor map, we found a significant between-groups difference in the area of the map sustained by a reduction in the post evaluation in controls (*T* = 0, *p* = 0.018, *r* = −0.79). No differences were found for the amplitude of the MEP and volume of the map. Figure 3 shows the results of MEP amplitude, area and volume of the motor map. However, we found a significant between-group effect in the shift of the CoG_*y*_ of the affected hemisphere (*U* = 6, *z* = −2.20, *p* = 0.028, *r* = −0.56) and in the unaffected hemisphere (*U* = 7, *z* = −2.06; *p* = 0.039, *r* = −0.55). Later comparisons only showed a significant posterior shift of the CoG_*y*_ of the affected hemisphere in patients (*T* = 0, *p* = 0.043, *r* = −0.64) and no changes were observed in the unaffected hemisphere (*T* = 1, *p* = 0.080, *r* = −0.50). No changes in CoG_*y*_ were observed in controls in both hemispheres (*p* > 0.5 for both comparisons). Figure 4 illustrates the displacement of the motor map in the affected hemisphere and Figure 5 shows the affected motor map of the cortical representation of the FDI muscle of the patients, before and after the therapy.

## Discussion

MST program improved motor function and quality of life of nine stroke patients accompanied by changes in the organization of the sensorimotor cortex, evidenced by a decreased AMT and a displacement of the motor map (CoG_*y*_).

Reduction of motor deficits was reflected by improvements in the ARAT, Arm Paresis Score and BBT test. These results are in congruence with previous studies validating the application of MST in stroke patients (Schneider et al., 2007, 2010; Altenmüller et al., 2009; Rojo et al., 2011; Amengual et al., 2013). As motor function was evaluated using motor test independent of and different from the music performance trained in the MST this argues clearly for generalization of the effects of MST to everyday movements. This is critical for a more widespread clinical application.

When playing the piano, patients have to perform fine movements with the affected hand to press the key in order to elicit the tone. This sensory feedback is thought to be essential in the success of the therapy (Rodríguez-Fornells et al., 2012) as patients have to readjust their movements in terms of temporal and spatial organization, coordination according to the rhythm, force and velocity. This idea is also in agreement with the essential role of audio-motor interactions in music processing (Zatorre et al., 2007) and the potential increase of plasticity when using multimodal learning paradigms (Lappe et al., 2008; Wan and Schlaug, 2010; Pantev and Herholz, 2011; Herholz and Zatorre, 2012). Furthermore, working with scales, tones and melodies allows a wide range of different sequences giving the task a greater variability. Such task variations enhance the creation of internal models which might be crucial to generalize motor skill learning to other different situations (Conditt et al., 1997).

While the improvement on motor tests seen here suggests a generalization to movements important for everyday tasks this conclusion needs to be substantiated. Gains in hand function in terms of greater strength and dexterity contribute to improvements in the functional use of the hand (Harris and Eng, 2007; Wolf et al., 2008) which in turn diminishes the level of disability and improves social participation (Carod-Artal et al., 2000; Wolf et al., 2008). However, improvements in motor function can take several months to become apparent in activities of daily living (Winstein et al., 2004). When validating new neurorehabilitation techniques it is crucial not only to test their efficacy, known as the degree to which the intervention affects functional outcome measures, but also to assess their effectiveness and how the therapy its influencing the quality of life (Nadeau, 2002). At this time, we only have results from the Stroke-Specific Quality of Life test where patients reported better quality of life. Future studies should include a follow-up evaluation and a compressive assessment of basic and instrumental activities of daily life, taking into account information from patients and caregivers.

Regarding to the motor threshold, AMT was decreased after the therapy showing a change in excitability of motor cortex. It has been postulated that motor thresholds could decrease during motor skill learning although this could be reestablished when the skill becomes overlearned (see for example, Pascual-Leone et al., 1995; Pearce et al., 2000). An inter-hemispheric asymmetry in motor thresholds has been described after unilateral damage to the corticomotor pathways (Groppa et al., 2012). In stroke, motor thresholds are increased in the affected hemisphere suggesting that cortical neurons increase their thresholds for excitation (Traversa et al., 1998; McConnell et al., 2001). RMT, measured at rest, depends on the excitability of presynaptic neurons to the corticospinal tract, the excitability of synapses at the cortex between excitatory inputs and corticospinal cells and the synaptic strength between the pyramidal neurons of the corticospinal tract and the motoneurons in the spinal cord (Talelli et al., 2006). Differently, AMT, measured when the muscle is contracted voluntarily by the subject, mainly depends on the membrane excitability, since synapses are pre-activaded due to the contraction of the muscle. Therefore, changes in AMT might explain specific regulation of the excitability at cortical level rather than spinal. A reduction in AMT therefore might signal functional recovery, as less intense stimulus-pulses elicit MEPs during tonic voluntary activity (Kobayashi and Pascual-Leone, 2003).

Differences in CoG_*y*_ showed changes of the motor map in terms of a posterior displacement in the vertical axis of the map. Functional motor recovery correlates with the reorganization of pyramidal neurons in the somatosensory cortex of the affected hemisphere (Pekna et al., 2012). Cortical motor output zones can expand to adjacent areas (Nudo and Milliken, 1996; Karl et al., 2001) and this displacement is associated with successful motor recovery (Jaillard et al., 2005). The posterior displacement of activation we encountered suggests reorganization of the motor representations. In their study validating the MST in a group of chronic patients, Amengual et al. (2013) reported a shift in CoG_*x*_, the coordinate of the CoG that represents the horizontal axis of the map, which demonstrates cortical reorganization after the MST program. Similar findings, albeit outside of the context of MST, have been reported by Pineiro et al. (2001) and Calautti et al. (2003). It has been seen that repetitive motor training, which is based on the repetition of an active motor sequence, does not produce functional reorganization of cortical maps (Jaillard et al., 2005) whereas motor skill acquisition, the process by which movements are executed more quickly and accurately with practice to accomplish a functional objective, leads to changes in the representation of a muscle in the motor cortex (Willingham, 1998; Nudo, 2006). This in turn suggests that the characteristics of MST are especially suited to induce motor plasticity.

Previous studies evaluating the size of the motor maps after training in healthy participants (Pascual-Leone et al., 1994, 1995) and in stroke patients (Liepert et al., 1998, 2000) described an increase in the number of cortical sites responding to a target muscle (Pekna et al., 2012). We did neither find changes in the area and volume of motor maps nor on the CSP and MEP amplitude measures after MST. One reason for this lack of findings in the area and volume of motor maps from patients might be the small sample size used. Future studies should therefore include more patients to confirm the MST effects on the reorganization of the sensorimotor cortex. Such larger scale studies should also differentiate between patients with cortical and subcortical lesions and lesions of the dominant vs. non-dominant hemisphere. Neuroimaging techniques, especially fMRI and brain connectivity analysis should also be included for complementing this information. Moreover, an important limitation of the study is the lack of a control group of patients. In the present study only a healthy control group was evaluated to reject the idea that changes in the excitability of the sensorimotor cortex are due to systematic time-dependent effects or possible brain changes over time (Johansen-Berg, 2012). Contrary to the expectations, we found that the area of the motor map was reduced in healthy participants. We could not clarify this point as we did not control if those participants did any type of motor learning during the pre and post evaluation that would explain changes in the area of the motor map (Pascual-Leone et al., 1994). For that reason, this effect limits the conclusions of the present study. Other studies have shown the benefits of MST comparing two groups of patients with different interventions, MST and conventional treatment (Schneider et al., 2007; Altenmüller et al., 2009). These designs are more appropriate and will be necessary in future studies when evaluating the effects of MST on the brain plasticity in stroke patients.

Finally, it is worth mentioning that music always entails emotion. Its emotional value might be important for the acceptance of the therapy program and its efficacy (Schneider et al., 2010). It has been suggested that neuroplasticity depends on the motivational value of the activity (Sanes and Donoghue, 2000). Therefore, the creation and validation of new therapies and their application into clinical practice should take emotional and motivational factors into account with the introduction of meaningful activities in the neurorehabilitation process.

### Conflict of interest statement

The authors declare that the research was conducted in the absence of any commercial or financial relationships that could be construed as a potential conflict of interest.

## References

[B1] Altenmüller E. Marco-Pallarés J. Münte T. F. Schneider S. (2009). Neural reorganization underlies improvement in stroke-induced motor dysfunction by music-supported therapy. Ann. N.Y. Acad. Sci. 1169, 395–405 10.1111/j.1749-6632.2009.04580.x 19673814

[B2] Amengual J. L. Rojo N. Veciana de las Heras M. Marco-Pallarés J. Grau-Sánchez J. Schneider S. (2013). Sensorimotor plasticity after music-supported therapy in chronic stroke patients revealed by transcranial magnetic stimulation. PLoS ONE 8:e61883 10.1371/journal.pone.0061883 23613966 PMC3629163

[B3] Amengual J. L. Valero Cabré A. Veciana de las Heras M. Rojo N. Froudist-Walsh S. Ripollés P. (2012). Prognostic value of cortically induced motor evoked activity by TMS in chronic stroke: caveats from a revealing single clinical case. BMC Neurol. 12:35 10.1186/1471-2377-12-35 22682434 PMC3411427

[B4] Bangert M. Peschel T. Schlaug G. Rotte M. Drescher D. Hinrichs H. (2006). Shared networks for auditory and motor processing in professional pianists: evidence from fMRI conjunction. Neuroimage 30, 917–926 10.1016/j.neuroimage.2005.10.044 16380270

[B5] Baumann S. Koeneke S. Schmidt C. F. Meyer M. Lutz K. Jancke L. (2007). A network for audio-motor coordination in skilled pianists and non-musicians. Brain Res. 1161, 65–78 10.1016/j.brainres.2007.05.045 17603027

[B6] Bengtsson S. L. Nagy Z. Skare S. Forsman L. Forssberg H. Ullén F. (2005). Extensive piano practicing has regionally specific effects on white matter development. Nat. Neurosci. 8, 1148–1150 10.1038/nn1516 16116456

[B7] Byrnes M. L. Thickbroom G. W. Phillips B. A. Wilson S. A. Mastaglia F. L. (1999). Physiological studies of the corticomotor projection to the hand after subcortical stroke. Clin. Neurophysiol. 110, 487–498 10.1016/S1388-2457(98)00044-3 10363772

[B8] Calautti C. Leroy F. Guincestre J. Y. Baron J. C. (2003). Displacement of primary sensorimotor cortex activation after subcortical stroke: a longitudinal PET study with clinical correlation. Neuroimage 19, 1650–1654 10.1016/S1053-8119(03)00205-2 12948719

[B9] Carod-Artal J. Egido J. A. González J. L. Varela de Seijas E. (2000). Quality of life among stroke survivors evaluated 1 year after stroke: experience of a stroke unit. Stroke 31, 2995–3000 10.1161/01.STR.31.12.2995 11108762

[B10] Carroll D. (1965). A quantitative test of upper extremity function. J. Chronic Dis. 18, 479–491 10.1016/0021-9681(65)90030-5 14293031

[B11] Chollet F. DiPiero V. Wise R. J. Brooks D. J. Dolan R. J. Frackowiak R. S. (1991). The functional anatomy of motor recovery after stroke in humans: a study with positron emission tomography. Ann. Neurol. 29, 63–71 10.1002/ana.410290112 1996881

[B12] Cohen J. (1992). A power primer. Psychol. Bull. 112, 155–159 10.1037/0033-2909.112.1.155 19565683

[B13] Conditt M. A. Gandolfo F. Mussa-Ivaldi F. A. (1997). The motor system does not learn the dynamics of the arm by rote memorization of past experiences. J. Neurophysiol. 78, 554–560 9242306 10.1152/jn.1997.78.1.554

[B14] Cramer S. C. Sur M. Dobkin B. H. O'Brien C. Sanger T. D. Trojanowski J. Q. (2011). Harnessing neuroplasticity for clinical applications. Brain 134, 1591–1609 10.1093/brain/awr039 21482550 PMC3102236

[B15] Dayan E. Cohen L. G. (2011). Neuroplasticity subserving motor skill learning. Neuron 72, 443–454 10.1016/j.neuron.2011.10.008 22078504 PMC3217208

[B16] Doyon J. Benali H. (2005). Reorganization and plasticity in the adult brain during learning of motor skills. Curr. Opin. Neurobiol. 15, 161–167 10.1016/j.conb.2005.03.004 15831397

[B17] Draganski B. Gaser C. Busch V. Schuierer G. Bogdahn U. May A. (2004). Neuroplasticity: changes in grey matter induced by training. Nature 427, 311–312 10.1038/427311a 14737157

[B18] Gaser C. Schlaug G. (2003). Brain structures differ between musicians and non-musicians. J. Neurosci. 23, 9240–9245 14534258 10.1523/JNEUROSCI.23-27-09240.2003 PMC6740845

[B19] Grefkes C. Nowak D. A. Eickhoff S. B. Dafotakis M. Küst J. Karbe H. (2008). Cortical connectivity after subcortical stroke assessed with functional magnetic resonance imaging. Ann. Neurol. 63, 236–246 10.1002/ana.21228 17896791

[B20] Groppa S. Oliviero A. Eisen A. Quartarone A. Cohen L. G. Mall V. (2012). A practical guide to diagnostic transcranial magnetic stimulation: report of an IFCN committee. Clin. Neurophysiol. 123, 858–882 10.1016/j.clinph.2012.01.010 22349304 PMC4890546

[B21] Hallett M. (2007). Transcranial magnetic stimulation: a primer. Neuron 55, 187–199 10.1016/j.neuron.2007.06.026 17640522

[B22] Harris J. E. Eng J. J. (2007). Paretic upper-limb strength best explains arm activity in people with stroke. Phys. Ther. 87, 88–97 10.2522/ptj.20060065 17179441

[B23] Herholz S. C. Zatorre R. J. (2012). Musical training as a framework for brain plasticity: behavior, function and structure. Neuron 76, 486–502 10.1016/j.neuron.2012.10.011 23141061

[B24] Hyde K. L. Lerch J. Norton A. Forgeard M. Winner E. Evans A. C. (2009). Musical training shapes structural brain development. J. Neurosci. 29, 3019–3025 10.1523/JNEUROSCI.5118-08.2009 19279238 PMC2996392

[B25] Jaillard A. Martin C. D. Garambois K. Lebas J. F. Hommel M. (2005). Vicarious function within the human primary motor cortex. A longitudinal fMRI stroke study. Brain 128, 1122–1138 10.1093/brain/awh456 15728652

[B26] Johansen-Berg H. (2012). The future of functionally-related structural change assessment. Neuroimage 62, 1293–1298 10.1016/j.neuroimage.2011.10.073 22056531 PMC3677804

[B27] Karl A. Birbaumer N. Lutzenberger W. Cohen L. G. Flor H. (2001). Reorganization of motor and somatosensory cortex in upper extremity amputees with phantom limb pain. J. Neurosci. 21, 3609–3618 11331390 10.1523/JNEUROSCI.21-10-03609.2001 PMC6762494

[B28] Karni A. Meyer G. Jezzard P. Adams M. M. Turner R. Ungerleider L. G. (1995). Functional MRI evidence for adult motor cortex plasticity during motor skill learning. Nature 377, 155–158 10.1038/377155a0 7675082

[B29] Kobayashi M. Pascual-Leone A. (2003). Transcranial magnetic stimulation in neurology. Lancet Neurol. 2, 145–156 10.1016/S1474-4422(03)00321-1 12849236

[B30] Kolb B. Whishaw I. Q. (1998). Brain plasticity and behavior. Ann. Rev. Psychol. 49, 43–64 10.1146/annurev.psych.49.1.43 9496621

[B31] Krakauer J. W. (2006). Motor learning: its relevance to stroke recovery and neurorehabilitation. Curr. Opin. Neurol. 19, 84–90 10.1097/01.wco.0000200544.29915.cc 16415682

[B32] Krakauer J. W. Ghilardi M. F. Ghez C. (1999). Independent learning of internal models for kinematic and dynamic control of reaching. Nat. Neurosci. 2, 1026–1031 10.1038/14826 10526344

[B33] Langhorne P. Bernhardt J. Kwakkel G. (2011). Stroke rehabilitation. Lancet 377, 1693–1702 10.1016/S0140-6736(11)60325-5 21571152

[B34] Lappe C. Herholz S. C. Trainor L. J. Pantev C. (2008). Cortical plasticity induced by short-term unimodal and multimodal musical training. J. Neurosci. 28, 9632–9639 10.1523/JNEUROSCI.2254-08.2008 18815249 PMC6671216

[B35] Liepert J. Bauder H. Wolfgang H. R. Miltner W. H. Taub E. Weiller C. (2000). Treatment–induced cortical reorganization after stroke in humans. Stroke 31, 1210–1216 10.1161/01.STR.31.6.1210 10835434

[B36] Liepert J. Miltner W. H. Bauder H. Sommer M. Dettmers C. Taub E. (1998). Motor cortex plasticity during constraint-induced movement therapy in stroke patients. Neurosci. Lett. 250, 5–8 10.1016/S0304-3940(98)00386-3 9696052

[B37] Liepert J. Restemeyer C. Kucinski T. Zittel S. Weiller C. (2005). Motor strokes: the lesion location determines motor excitability changes. Stroke 36, 2648–2653 10.1161/01.STR.0000189629.10603.02 16269647

[B38] Lotze M. Scheler G. Tan H.-R. M. Braun C. Birbaumer N. (2003). The musician's brain: functional imaging of amateurs and professionals during performance and imagery. Neuroimage 20, 1817–1829 10.1016/j.neuroimage.2003.07.018 14642491

[B39] Lyle R. C. (1981). A performance test for assessment of upper limb function in physical rehabilitation treatment and research. Int. J. Rehabil. Res. 4, 483–492 10.1097/00004356-198112000-00001 7333761

[B40] Mathiowetz V. Volland G. Kashman N. Weber K. (1985). Adult norms for the box and block test of manual dexterity. Am. J. Occup. Ther. 39, 386–391 10.5014/ajot.39.6.386 3160243

[B41] McConnell K. A. Nahas Z. Shastri A. Lorberbaum J. P. Kozel F. A. Bohning D. E. (2001). The transcranial magnetic stimulation motor threshold depends on the distance from coil to underlying cortex: a replication in healthy adults comparing two methods of assessing the distance to cortex. Biol. Psychiatry 49, 454–459 10.1016/S0006-3223(00)01039-8 11274657

[B42] Meister I. G. Krings T. Foltys H. Boroojerdi B. Müller M. Töpper R. (2004). Playing piano in the mind: An fMRI study on music imagery and performance in pianists. Cogn. Brain Res. 19, 219–228 10.1016/j.cogbrainres.2003.12.005 15062860

[B43] Muellbacher W. Richards C. Ziemann U. Wittenberg G. Weltz D. Boroojerdi B. (2002). Improving hand function in chronic stroke. Arch. Neurol. 59, 1278–1282 10.1001/archneur.59.8.1278 12164724

[B44] Murase N. Duque J. Mazzocchio R. Cohen L. G. (2004). Influence of interhemispheric interactions on motor function in chronic stroke. Ann. Neurol. 55, 400–409 10.1002/ana.10848 14991818

[B45] Nadeau S. E. (2002). A paradigm shift in neurorehabilitation. Lancet Neurol. 1, 126–130 10.1016/S1474-4422(02)00044-3 12849517

[B46] Nudo R. J. (2006). Plasticity. NeuroRx 3, 420–427 10.1016/j.nurx.2006.07.006 17012055 PMC3593404

[B47] Nudo R. J. Milliken G. W. (1996). Reorganization of movement representations in primary motor cortex following focal ischemic infarcts in adult squirrel monkeys. J. Neurophysiol. 75, 2144–2149 8734610 10.1152/jn.1996.75.5.2144

[B48] Nudo R. J. Wise B. M. Sifuentes F. Milliken G. W. (1996). Neural substrates for the effects of rehabilitative training on motor recovery after ischemic infarct. Science 21, 1791–1794 10.1126/science.272.5269.1791 8650578

[B49] Pantev C. Herholz S. C. (2011). Plasticity of the human auditory cortex related to musical training. Neurosci. Biobehav. Rev. 35, 2140–2154 10.1016/j.neubiorev.2011.06.010 21763342

[B50] Parker V. M. Wade D. T. Langton-Hewer R. (1986). Loss of arm function after stroke: measurement, frequency and recovery. Int. Rehabil. Med. 8, 69–73 3804600 10.3109/03790798609166178

[B51] Pascual-Leone A. Grafman J. Hallett M. (1994). Modulation of cortical output maps during development of implicit and explicit knowledge. Science 263, 1287–1289 10.1126/science.8122113 8122113

[B52] Pascual-Leone A. Nguyet D. Cohen L. G. Brasil-Nieto J. P. Cammarota A. Hallett M. (1995). Modulation of muscle responses evoked by transcranial magnetic stimulation during the acquisition of new fine motor skills. J. Neurophysiol. 74, 1037–1045 7500130 10.1152/jn.1995.74.3.1037

[B53] Pearce A. J. Thickbroom G. W. Byrnes M. L. Mastaglia F. L. (2000). Functional reorganization of the corticomotor projection to the hand in skilled racquet players. Exp. Brain Res. 130, 238–243 10.1007/s002219900236 10672477

[B54] Pekna M. Pekny M. Nilsson M. (2012). Modulation of neural plasticity as a basis for stroke rehabilitation. Stroke 43, 2819–2828 10.1161/STROKEAHA.112.654228 22923444

[B55] Penhune V. B. Steele C. J. (2012). Parallel contributions of cerebellar, striatal and M1 mechanisms to motor sequence learning. Behav. Brain Res. 226, 579–591 10.1016/j.bbr.2011.09.044 22004979

[B56] Pineiro R. Pendlebury S. Johansen-Berg H. Matthews P. M. (2001). Functional MRI detects posterior shifts in primary sensorimotor cortex activation after stroke: evidence of local adaptive reorganization. Stroke 32, 1134–1139 10.1161/01.STR.32.5.1134 11340222

[B57] Rathore S. S. Hinn A. R. Cooper L. S. Tyroler H. A. Rosamond W. D. (2002). Characterization of incident stroke signs and symptoms: findings from the atherosclerosis risk in communities study. Stroke 33, 2718–2721 10.1161/01.STR.0000035286.87503.31 12411667

[B58] Rodríguez-Fornells A. Rojo N. Amengual J. L. Ripollés P. Altenmüller E. Münte T. F. (2012). The involvement of audio-motor coupling in the music-supported therapy applied to stroke patients. Ann. N.Y. Acad. Sci. 1252, 282–293 10.1111/j.1749-6632.2011.06425.x 22524370

[B59] Rojo N. Amengual J. L. Juncadella M. Rubio F. Camara E. Marco-Pallarés J. (2011). Music-supported therapy induces plasticity in the sensorimotor cortex in chronic stroke: a single-case study using multimodal imaging (fMRI-TMS). Brain Inj. 25, 787–793 10.3109/02699052.2011.576305 21561296

[B60] Rossi S. Hallett M. Rossini P. M. Pascual-Leone A. the Safety of TMS Consensus Group. (2009). Safety, ethical considerations, and application guidelines for the use of transcranial magnetic stimulation in clinical practice research. Clin. Neurophysiol. 120, 2008–2039 10.1016/j.clinph.2009.08.016 19833552 PMC3260536

[B61] Rossini P. M. Barker A. T. Berardelli A. Caramia M. D. Caruso G. Cracco R. Q. (1994). Non-invasive electrical and magnetic stimulation of the brain, spinal cord and roots: basic principles and procedures for routine clinical application. Report of an IFCN committee. Electroencephalogr. Clin. Neurophysiol. 91, 79–92 10.1016/0013-4694(94)90029-9 7519144

[B62] Sanes J. N. Donoghue J. P. (2000). Plasticity and primary motor cortex. Annu. Rev. Neurosci. 23, 393–415 10.1146/annurev.neuro.23.1.393 10845069

[B63] Särkämö T. Soto D. (2012). Music listening after stroke: beneficial effects and potential neural mechanisms. Ann. N.Y. Acad. Sci. 1252, 266–281 10.1111/j.1749-6632.2011.06405.x 22524369

[B64] Särkämö T. Tervaniemi M. Laitinen S. Forsblom A. Soinila S. Mikkonen M. (2008). Music listening enhances cognitive recovery and mood after middle cerebral artery stroke. Brain 131, 866–876 10.1093/brain/awn013 18287122

[B65] Schneider S. Münte T. F. Rodríguez-Fornells A. Sailer M. Altenmüller E. (2010). Music-supported training is more efficient than functional motor training for recovery of fine motor skills in stroke patients. Music Percept. 27, 271–280 10.1525/mp.2010.27.4.271

[B66] Schneider S. Schönle P. W. Altenmüller E. Münte T. F. (2007). Using musical instruments to improve motor skill recovery following a stroke. J. Neurol. 254, 1339–1346 10.1007/s00415-006-0523-2 17260171

[B67] Steele C. J. Bailey J. A. Zatorre R. J. Penhune V. B. (2013). Early musical training and white-matter plasticity in the corpus callosum: evidence for a sensitive period. J. Neurosci. 33, 1282–1290 10.1523/JNEUROSCI.3578-12.2013 23325263 PMC6704889

[B68] Talelli P. Greenwood R. J. Rothwell J. C. (2006). Arm function after stroke: neurophysiological correlates and recovery mechanisms assessed by transcranial magnetic stimulation. Clin. Neurophysiol. 117, 1641–1659 10.1016/j.clinph.2006.01.016 16595189

[B69] Taub E. Miller N. E. Novack T. A. Cook E. W. 3rd. Fleming W. C. Nepomuceno C. S. (1993). Technique to improve chronic motor deficit after stroke. Arch. Phys. Med. Rehabil. 74, 347–354 8466415

[B70] Taub E. Uswatte G. Elbert T. (2002). New treatments in neurorehabilitation founded on basic research. Nat. Rev. Neurosci. 3, 228–236 10.1038/nrn754 11994754

[B71] Traversa R. Cicinelli P. Pasqualetti P. Filippi M. Rossini P. M. (1998). Follow-up of interhemispheric differences of motor evoked potentials from the ‘affected’ and ‘unaffected’ hemispheres in human stroke. Brain Res. 803, 1–8 10.1016/S0006-8993(98)00505-8 9729235

[B72] Wade D. T. Langton-Hewer R. Wood V. A. Skilbeck C. E. Ismail H. M. (1983). The hemiplegic arm after stroke: measurement and recovery. J. Neurol. Neurosurg. Psychiatry 46, 521–524 10.1136/jnnp.46.6.521 6875585 PMC1027442

[B73] Wan C. Y. Schlaug G. (2010). Music making as a tool for promoting brain plasticity across the life span. Neuroscientist 16, 566–577 10.1177/1073858410377805 20889966 PMC2996135

[B74] Warlow C. P. van Gijn J. Dennis M. S. Wardlaw J. M. Bamford J. M. Hankey G. J. (2008). Stroke: Practical Management. Oxford: Blackwell Publishing

[B75] Wassermann E. M. Epstein C. M. Ziemann U. Walsh V. Paus T. Lisanby S. H. (2008). The Oxford Handbook of Trancranial Stimulation. New York: Oxford University Press

[B76] Wassermann E. M. McShane L. M. Hallett M. Cohen L. G. (1992). Noninvasive mapping of muscle representations in human motor cortex. Electroencephalogr. Clin. Neurophysiol. 85, 1–8 10.1016/0168-5597(92)90094-R 1371738

[B77] Williams L. S. Weinberger M. Harris L. E. Clark D. O. Biller J. (1999). Development of a stroke-specific quality of life scale. Stroke 30, 1362–1369 10.1161/01.STR.30.7.1362 10390308

[B78] Willingham D. B. (1998). A neuropsychological theory of motor skill learning. Psychol. Rev. 105, 558–584 10.1037/0033-295X.105.3.558 9697430

[B79] Wilson S. A. Thickbroom G. W. Mastaglia F. L. (1993). Transcranial magnetic stimulation mapping of the motor cortex in normal subjects. The representation of two intrinsic hand muscles. J. Neurol. Sci. 118, 134–144 10.1016/0022-510X(93)90102-5 8229061

[B80] Winstein C. J. Rose D. K. Tan S. M. Lewthwaite R. Chui H. C. Azen S. P. (2004). A randomized controlled comparison of upper-extremity rehabilitation strategies in acute stroke: a pilot study of immediate and long-term outcomes. Arch. Phys. Med. Rehabil. 85, 620–628 10.1016/j.apmr.2003.06.027 15083439

[B81] Wolf S. L. Winstein C. J. Miller J. P. Thompson P. A. Taub E. Uswatte G. (2008). Retention of upper limb function in stroke survivors who have received constraint-induced movement therapy: the EXCITE randomized trial. Lancet Neurol. 7, 33–40 10.1016/S1474-4422(07)70294-6 18077218 PMC2329576

[B82] World Health Organization. (2003). World Health Report. Geneva: World Health Organization.

[B83] Zatorre R. J. (2003). Music and the brain. Ann. N.Y. Acad. Sci. 999, 4–14 10.1196/annals.1284.001 14681113

[B84] Zatorre R. J. Chen J. L. Penhune V. B. (2007). When the brain plays music: auditory-motor interactions in music perception and production. Nat. Rev. Neurosci. 8, 547–558 10.1038/nrn2152 17585307

